# Utilization of neoadjuvant chemotherapy in high‐risk, node‐negative early breast cancer

**DOI:** 10.1002/cam4.4517

**Published:** 2022-01-05

**Authors:** Ipshita Prakash, N. Ben Neely, Samantha M. Thomas, Sarah Sammons, Rachel C. Blitzblau, Gayle A. DiLalla, Terry Hyslop, Carolyn S. Menendez, Jennifer K. Plichta, Laura H. Rosenberger, Oluwadamilola M. Fayanju, E. Shelley Hwang, Rachel A. Greenup

**Affiliations:** ^1^ Department of Surgery Jewish General Hospital McGill University Montreal Quebec Canada; ^2^ Duke Department of Biostatistics & Bioinformatics Durham North Carolina USA; ^3^ Duke Cancer Institute Durham North Carolina USA; ^4^ Department of Medicine Duke University School of Medicine Durham North Carolina USA; ^5^ Department of Radiation Oncology Duke University School of Medicine Durham North Carolina USA; ^6^ Department of Surgery Duke University School of Medicine Durham North Carolina USA; ^7^ Department of Population Health Sciences Duke University School of Medicine Durham North Carolina USA; ^8^ Division of Endocrine and Oncologic Surgery Department of Surgery Perelman School of Medicine The University of Pennsylvania Philadelphia Pennsylvania USA; ^9^ Rena Rowan Breast Center Abramson Cancer Center Philadelphia Pennsylvania USA; ^10^ Yale Department of Surgery Yale School of Medicine New Haven Connecticut USA; ^11^ Yale Cancer Outcomes, Public Policy and Effectiveness Research Center (COPPER) Yale School of Medicine and Yale Cancer Center New Haven Connecticut USA

**Keywords:** breast cancer, cancer management, clinical management, neoadjuvant chemotherapy, surgical oncology

## Abstract

**Background:**

Controversy exists regarding the optimal sequence of chemotherapy among women with operable node‐negative breast cancers with high‐risk tumor biology. We evaluated national patterns of neoadjuvant chemotherapy (NACT) use among women with early‐stage HER2+, triple‐negative (TNBC), and high‐risk hormone receptor‐positive (HR+) invasive breast cancers.

**Methods:**

Women ≥18 years with cT1‐2/cN0 HER2+, TNBC, or high recurrence risk score (≥31) HR+ invasive breast cancers who received chemotherapy were identified in the National Cancer Database (2010–2016). Cochran‐Armitage and logistic regression examined temporal trends and likelihood of undergoing NACT versus adjuvant chemotherapy based on patient age and molecular subtype.

**Results:**

Overall, 96,622 patients met study criteria; 25% received NACT and 75% underwent surgery first, with comparable 5‐year estimates of overall survival (0.90, 95% CI 0.892–0.905 vs 0.91, 95% CI 0.907–0.913). During the study period, utilization of NACT increased from 14% to 36% and varied according to molecular subtype (year*molecular subtype *p* < 0.001, *p*‐corrected < 0.001). Women with HER2+ (OR 4.17, 95% CI 3.70–4.60, *p* < 0.001, *p*‐corrected < 0.001) and TNBC (OR 3.81, 95% CI 3.38–4.31, *p* < 0.001, *p*‐corrected < 0.001) were more likely to receive NACT over time, without a change in use among those with HR+ disease (OR 1.58, 95% CI 0.88–2.87, *p* = 0.13, *p*‐corrected = 0.17).

**Conclusion:**

Among women with early‐stage triple‐negative and HER2+ breast cancers, utilization of NACT increased over time, a trend that correlates with previously reported improved rates of pCR and options post‐neoadjuvant treatment with residual disease. Future research is needed to better understand multidisciplinary decisions for NACT and implications for breast cancer patients.

## Graphical abstract

Among high‐risk tumor biology, the utilization of neoadjuvant chemotherapy (NACT) has increased over time. NACT is paradoxically associated with higher rates of contralateral prophylactic mastectomy and lower rates of breast conservation.

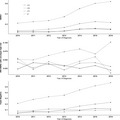

## 1 INTRODUCTION

In the setting of early‐stage breast cancer, indications for and utilization of neoadjuvant chemotherapy (NACT) have grown dramatically over the past several decades. The landmark NSABP B‐18, NSABP B‐27, and EORTC 10902 clinical trials first demonstrated that preoperative chemotherapy was associated with improved eligibility for breast conservation and equivalent oncologic outcomes, without a detriment in disease‐specific or overall survival.^1^, ^2^, ^3^ A more recent meta‐analysis from the Early Breast Cancer Trialists’ Collaborative Group (EBCTCG) included patient‐level data from ten randomized clinical trials and unexpectedly demonstrated that utilization of NACT was associated with a higher risk of local recurrence, with an absolute increase of 5.5% at 15 years.^4^

Notably, the aforementioned studies did not account for the evolving prognostic and predictive value of pathologic complete response (pCR). Contemporary use of NACT allows for an in vivo assessment of tumor response, the potential to deescalate surgical treatment, and the invaluable opportunity to deliver targeted therapies in the setting of residual disease.^1^, ^5^, ^6^, ^7^ pCR correlates with breast cancer prognosis and varies by tumor subtype, with the greatest response occurring in higher risk tumor biology [hormone receptor‐positive (HR+)/HER2−, 7%–15%; hormone receptor‐positive (HR+)/HER2+, 30%–40%; hormone receptor‐negative (HR−)/HER2+, 50%–70%; triple‐negative breast cancer (TNBC), 25%–30%).^7^, ^8^, ^9^, ^10^, ^11^, ^12^ The absence of pCR suggests biologic resistance and identifies a patient population disproportionately at risk. Most recently, escalation of systemic therapy with post‐neoadjuvant treatments (i.e., trastuzamab emtansine, capecitabine) has demonstrated improved survival for women with residual disease and has undoubtedly influenced patterns of clinical care.^13^, ^14^ Current guidelines recommend consideration of NACT in the early‐stage setting to improve eligibility for breast conservation *or* in women with tumors ≥2 cm with triple‐negative or HER2+ subtypes.^15^, ^16^, ^17^ However, multidisciplinary consensus remains less clear regarding the appropriate sequence of chemotherapy in women with smaller tumors and/or HR+ disease. Women with clinical T1N0 tumors have an excellent prognosis at baseline, with long‐term disease‐specific survival exceeding 95%, and may therefore derive less benefit from NACT.^18^, ^19^ Conversely, information regarding pathologic response to treatment has the potential to alter recommendations for adjuvant systemic and radiation therapy. In the context of this uncertainty, we sought to examine national trends in NACT utilization among early‐stage high‐risk breast cancer.

## 2 METHODS

### 2.1 Study population

Using the National Cancer Database (2010–2016), we identified female patients ≥18 years old with a clinical T1 or T2 node‐negative (cT1‐2N0), histologically‐confirmed ductal or lobular invasive breast cancer who received chemotherapy. Patients were excluded if they did not undergo both chemotherapy and surgery or had unknown/missing data for treatment and/or timing of treatment.

### 2.2 Statistical analysis

Patients were categorized by treatment sequence as surgery first (received adjuvant chemotherapy) or neoadjuvant chemotherapy (NACT) and patient characteristics between groups were compared. Continuous variables are presented as medians (Q1, Q3), and differences were compared using the *t*‐test when the assumption of normality was satisfied; otherwise, the Wilcoxon rank‐sum test was used. Categorical variables are presented as counts (proportions). Differences were compared using the *χ* ^2^ test. For all analyses, age was divided as 18–45, 46–64, and 65+ years. For the purpose of our study, high‐risk molecular subtype was categorized as HER2+, TNBC, and hormone receptor‐positive (HR+/HER2−) with recurrence risk score of ≥31.^20^ Of note, the high‐risk recurrence score cut‐off reflected national standards during the study period. Women with hormone receptor‐positive breast cancers who did not have evidence of OncotypeDx testing or had low‐intermediate recurrence risk scores were excluded. This reflects our intent to study breast cancer patients in whom chemotherapy was recommended based on biologic risk who would presumably have been eligible for systemic therapy in either the neoadjuvant or adjuvant settings. To examine the utilization of NACT over time, the proportion of NACT recipients by year of diagnosis was plotted in each subgroup. Within each subgroup, the Cochran‐Armitage trend test was conducted.

Logistic regression was used to estimate the association between treatment sequence (i.e., receipt of NACT vs. surgery first) and year of diagnosis both in univariate analyses and after adjusting for known covariates including facility type, facility location, insurance status, ductal vs. lobular histology, endocrine therapy, radiation therapy, race/ethnicity, tumor size, tumor grade, surgery type, and the Charlson‐Deyo co‐morbidity score. Subsequent models that included year of diagnosis*age and year of diagnosis*molecular subtype interaction terms were used to determine if that association varied by age or molecular subtype. Year of diagnosis was treated as a categorical variable to allow the change in the odds of treatment to vary flexibly across years, and the following levels were considered in the modeling: 2010, 2011, 2012, 2013, 2014, 2015, and 2016. All models utilized the generalized estimating equations (GEE) framework and a compound symmetric correlation structure to account for the correlation of patients treated at the same facility.

A sub‐group sensitivity analysis was performed to examine the use of NACT based on sub‐category of clinical T stage as defined by the American Joint Committee on Cancer (AJCC) 8th edition (cT1a, cT1b, cT1c, and cT2).^21^ The study population within each molecular subtype was subdivided by size, and the interaction between tumor size and categorical year of diagnosis on NACT usage was modeled.

Overall survival estimates were derived using the Kaplan‐Meier method. Estimates were stratified by indication of surgery first or NACT. Survival estimates at 5 years are reported along with 95% confidence intervals.

False Discovery Rate (FDR) family‐wise adjustments for multiple comparisons were made for the Cochran‐Armitage analysis and each unadjusted and adjusted model. Only patients with complete data for all covariates were included for each analysis, and effective sample sizes are included for all tables and figures. All statistical analyses were conducted using SAS, version 9.4 (SAS Institute, Cary, NC) and R version 3.6.1.

## 3 RESULTS

### 3.1 Baseline and treatment characteristics

**FIGURE 1:**
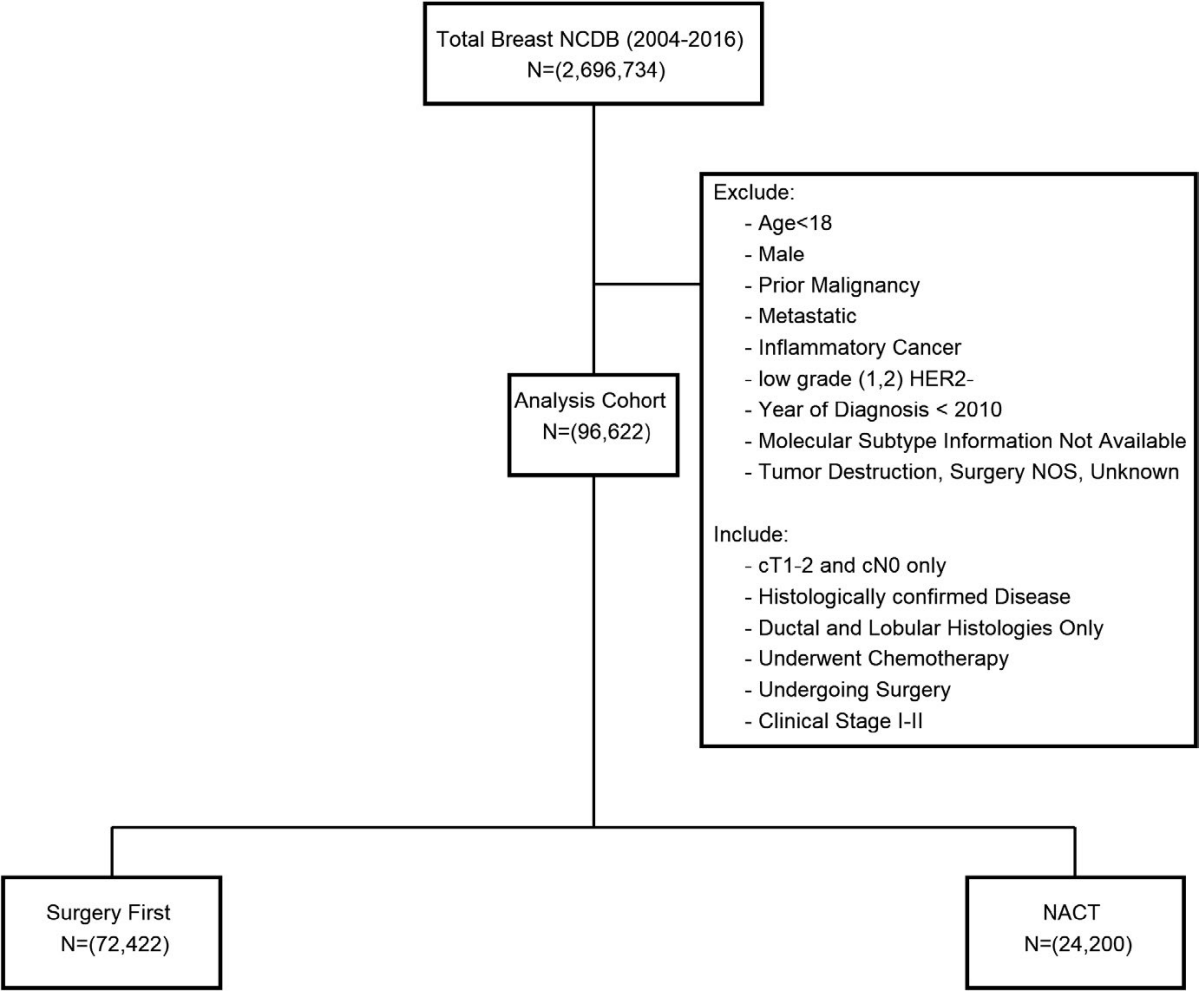
Female patients with early‐stage breast cancer, National Cancer Database (2010–2016)

96,622 patients met our inclusion criteria (Figure 1; Table 1). The vast majority of patients underwent surgery first (75%; *n* = 72,422) while 25% (*n* = 24,200) received NACT. When compared to the surgery first group, women who received NACT were younger (18–45 years: 31.6% vs 17.8%, *p* < 0.001) and had higher tumor stage (cT2: 69.8% vs 31.9%, *p* < 0.001). The 5‐year estimates of overall survival were comparable between women receiving NACT and those undergoing surgery first (0.90, 95% CI 0.892–0.905 vs 0.91, 95% CI 0.907–0.913). The distribution of tumor grade was also similar between the two groups (grade 1: 2.2% vs 2.3%; grade 2: 22.2% vs 19.5%; grade 3: 75.6% vs 78.2%, *p* < 0.001). Additionally, there were similar rates of HER2+ (56.9% vs 52.6%) and triple‐negative (41.7% vs 38.4%) subtypes, and a lower proportion of high‐risk HR+/HER2‐ breast cancers (1.4% vs 9.1%). Notably, women who received NACT were more likely to have private insurance (70.7% vs 63.2%, *p* < 0.001) and less likely to have government‐issued insurance (26.7% vs 34.9%, *p* < 0.001) when compared to those undergoing surgery first. In addition, receipt of NACT was associated with lower rates of breast‐conserving surgery (63.1% vs 51.2%) and higher rates of contralateral prophylactic mastectomy (14.8% vs 27.3%) compared to receipt of chemotherapy in the adjuvant setting.

**TABLE 1:** Patient demographics and tumor characteristics, National Cancer Database (2010–2016)

	Total (*N* = 96,622)	NACT (*N* = 24,200)	Surgery first (*N* = 72,422)	*p*‐Value
Age group				<0.001
≥18 to ≤45	20,522 (21.2%)	7,640 (31.6%)	12,882 (17.8%)	
>45 to ≤64	53,272 (55.1%)	12,923 (53.4%)	40,349 (55.7%)	
≥65	22,828 (23.6%)	3,637 (15.0%)	19,191 (26.5%)	
Year of diagnosis				<0.001
2010	10,820 (11.2%)	1,483 (6.1%)	9,337 (12.9%)	
2011	11,910 (12.3%)	1,876 (7.8%)	10,034 (13.9%)	
2012	12,683 (13.1%)	2,280 (9.4%)	10,403 (14.4%)	
2013	13,906 (14.4%)	2,907 (12.0%)	10,999 (15.2%)	
2014	15,365 (15.9%)	4,441 (18.4%)	10,924 (15.1%)	
2015	16,101 (16.7%)	5,524 (22.8%)	10,577 (14.6%)	
2016	15,837 (16.4%)	5,689 (23.5%)	10,148 (14.0%)	
Molecular subtype				<0.001
HER2+	51,846 (53.7%)	13,775 (56.9%)	38,071 (52.6%)	
HR+ with high‐risk oncotype	6,918 (7.2%)	343 (1.4%)	6,575 (9.1%)	
Triple negative	37,858 (39.2%)	10,082 (41.7%)	27,776 (38.4%)	
Charleson‐Deyo comorbidity score				<0.001
0	82,272 (85.1%)	21,454 (88.7%)	60,818 (84.0%)	
1	11,740 (12.2%)	2,244 (9.3%)	9,496 (13.1%)	
2	1,967 (2.0%)	366 (1.5%)	1,601 (2.2%)	
3	643 (0.7%)	136 (0.6%)	507 (0.7%)	
Tumor size (cm)—Median (IQR)	1.9 (1.3–2.7)	2.5 (1.7–3.3)	1.8 (1.2–2.5)	<0.0001
AJCC staging				<0.001
cT1mi	331 (0.3%)	25 (0.1%)	306 (0.4%)	
cT1	10,975 (11.4%)	953 (3.9%)	10,022 (13.8%)	
cT1a	2,852 (3.0%)	305 (1.3%)	2,547 (3.5%)	
cT1b	10,980 (11.4%)	924 (3.8%)	10,056 (13.9%)	
cT1c	31,508 (32.6%)	5,095 (21.1%)	26,413 (36.5%)	
cT2	39,976 (41.4%)	16,898 (69.8%)	23,078 (31.9%)	
Surgery type				<0.001
BCS	58,057 (60.1%)	12,387 (51.2%)	45,670 (63.1%)	
CPM	17,292 (17.9%)	6,597 (27.3%)	10,695 (14.8%)	
Mastectomy	21,266 (22.0%)	5,210 (21.5%)	16,056 (22.2%)	
Radiation therapy				<0.001
No	35,111 (36.4%)	10,480 (43.4%)	24,631 (34.1%)	
Yes	61,262 (63.6%)	13,655 (56.6%)	47,607 (65.9%)	
Facility type				<0.001
Academic	31,269 (32.4%)	8,539 (35.3%)	22,730 (31.4%)	
Community	8,663 (9.0%)	1,728 (7.1%)	6,935 (9.6%)	
Comprehensive	42,398 (43.9%)	9,915 (41.0%)	32,483 (44.9%)	
Integrated Network	14,292 (14.8%)	4,018 (16.6%)	10,274 (14.2%)	
Insurance status				<0.001
Government	31,366 (32.8%)	6,382 (26.7%)	24,984 (34.9%)	
Not Insured	2,036 (2.1%)	621 (2.6%)	1,415 (2.0%)	
Private	62,201 (65.1%)	16,915 (70.7%)	45,286 (63.2%)	
Race/ethnicity				<0.001
Hispanic	5,704 (6.1%)	1,640 (7.0%)	4,064 (5.8%)	
Non‐Hispanic black	14,268 (15.2%)	3,552 (15.1%)	10,716 (15.3%)	
Non‐Hispanic white	69,221 (73.9%)	17,060 (72.3%)	52,161 (74.4%)	
Other	4,478 (4.8%)	1,332 (5.6%)	3,146 (4.5%)	

Note

Continuous differences were compared using the *t*‐test when the assumption of normality was satisfied; otherwise, the Wilcoxon rank‐sum test was used. Categorical variables are presented as counts (proportions). Categorical differences were compared using the *χ* ^2^ test.

Percentages may not add up to 100 due to rounding or missing errors.

Abbreviations: BCS, breast conserving surgery; CPM, contralateral prophylactic mastectomy; NACT, neoadjuvant chemotherapy only; NOS, not otherwise specified.

### 3.2 Temporal trends

**FIGURE 2:**
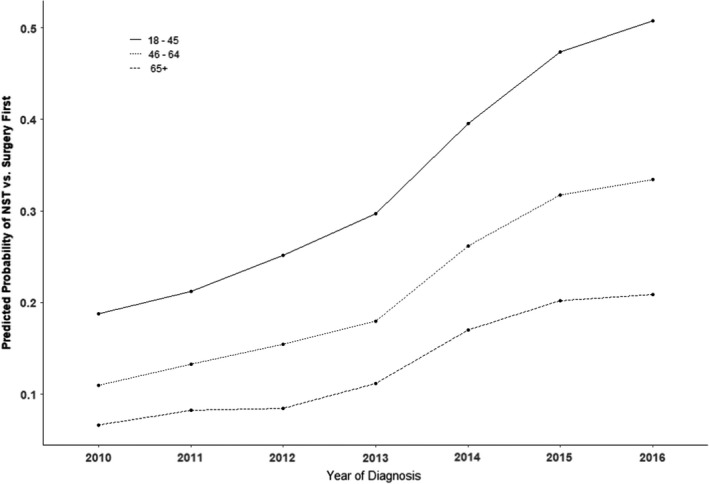
Predicted probability of NACT vs. surgery first by age, 2010–2016 (*N* = 96,622)

**FIGURE 3:**
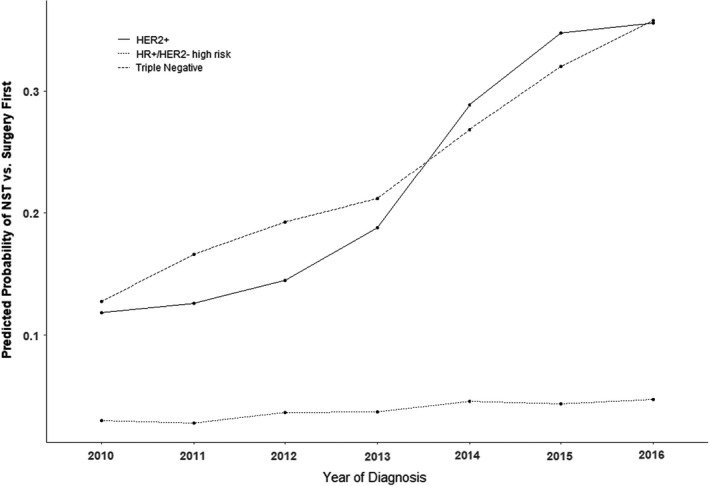
Predicted probability of NACT vs. surgery first by tumor subtype, 2010–2016 (*N* = 96,622)

From 2010 to 2016, the overall use of NACT increased significantly from 14% to 36% (Cochran‐Armitage *p* < 0.0001, *p*‐corrected < 0.001). Although the absolute proportion of women undergoing NACT was higher in younger women, there was no significant difference in the rate of increase over time according to patient age (year*age *p* = 0.29, *p*‐corrected = 0.32; Figure 2). However, temporal trends of NACT utilization varied according to molecular subtype during the study period (year*molecular subtype *p* < 0.001, *p*‐corrected < 0.001). When comparing data from 2016 vs 2010 (Figure 3), NACT use in patients with HER2+ breast cancer (OR 4.17 [95% CI 3.70–4.60]), and TNBC (OR 3.81 [95% CI 3.38–4.31]) increased significantly over time. However, there was no significant increase for women with high‐risk HR+/HER2− breast cancer (OR 1.58 [95% CI 0.88–2.87] *p* = 0.13, *p*‐corrected = 0.17). Similar conclusions were found after adjusting for potential confounders.

**FIGURE 4:**
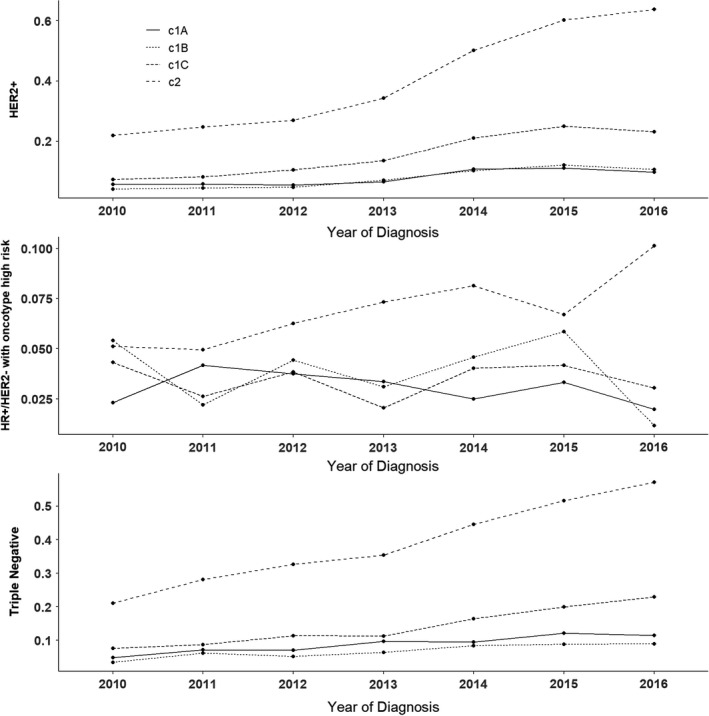
Predicted probability of NACT vs. surgery first by T‐stage (HER2+: *N* = 51,846; HR+: *N* = 6,918; TNBC: *N* = 37,858)

Subgroup analyses were performed according to the anatomical AJCC clinical T‐stage (cT1a, cT1b, cT1c, cT2) within each molecular subtype **(**Figure 4) to compare receipt of NACT vs surgery first between 2016 and 2010. Greater increases in NACT use were seen among women with cT2 HER2+ tumors (OR 6.26, 95% CI 5.41–7.25) than HER2+ cT1a (OR 1.79, 95% CI 1.18–2.70), cT1b (OR 2.82, 95% CI 1.74–4.58), and cT1c (OR 3.91, 95% CI 3.10–4.91; all *p* < 0.01). After adjusting for multiple comparisons, the HER2+ cT1a association attenuated and we found no evidence of an increase over time (*p*‐corrected 0.07), yet all other clinical T‐stage associations remained significant (*p*‐corrected < 0.001). Despite greater use of NACT in later years, the rate of change based on tumor size was not significant among women with TNBC (*p* = 0.06, *p*‐corrected = 0.08) or HR+/HER2− breast cancers (*p* = 0.22, *p*‐corrected = 0.26).

## 4 DISCUSSION

In early‐stage breast cancer, indications for neoadjuvant chemotherapy (NACT) have expanded beyond the original intent of improving eligibility for breast conservation to now include in vivo assessment of tumor response, prognostication, and identification of chemoresistance that may benefit from escalation of systemic therapy. Our study reviewed a contemporary cohort of women with high‐risk early breast cancer, demonstrating a nearly three‐fold increase in the overall use of NACT during the study period (2010–2016). In this population of individuals with T1‐2N0 breast cancers necessitating chemotherapy, delivery in the preoperative setting was independently associated with HER2+ and triple‐negative biology and federal approval for drug use. These findings are likely explained by greater collective understanding of pCR as a surrogate marker for long‐term outcomes and increasing awareness of post‐neoadjuvant treatment options.^7^ Among HER2+ breast cancers, we observed the steepest increase in NACT utilization between 2013 and 2014. This is best explained by Federal Drug Agency (FDA) approval of pertuzumab in the neoadjuvant setting (September 2013) following publication of the NeoSphere trial, which demonstrated increased pCR rates with combination neoadjuvant pertuzumab and trastuzamab.^22^ Notably, use of NACT did not significantly change over time in women with HR+/HER2− biology. Similar to previously published literature, we found that NACT was paradoxically associated with lower rates of breast conserving surgery and increased rates of contralateral prophylactic mastectomy.^23^

Recommendations for NACT are seemingly uncomplicated in women with high‐risk tumor biology who will receive clear benefit (i.e., large tumor‐to‐breast ratio, axillary node‐positive disease, HER2+ or TNBC with tumors ≥2 cm). Yet, multidisciplinary consensus around chemotherapy sequence may be difficult in women for whom NACT is unlikely to alter the surgical plan, in those with a lower chance of attaining pCR, and in whom surgical pathology results may contribute to tailored treatment recommendations. In the context of this uncertainty, we aimed to evaluate national patterns of NACT among women with high‐risk early‐stage breast cancer.

Pathologic response to NACT is now critical in identifying patients who may benefit from additional post‐neoadjuvant therapy. Recent landmark clinical trials (KATHERINE; CREATE‐X) have demonstrated significantly improved survival among HER2+ and TNBC patients with residual disease who received adjuvant trastuzumab emtansine (T‐DM1) and capecitabine.^13^, ^14^ While the effect of the CREATE‐X and KATHERINE trials was not captured in our study, these trials will presumably result in further expansion of NACT among women with early‐stage breast cancers. Rapidly evolving translational data and systemic therapy options arguably complicate the clinical management of breast cancer patients with small, node‐negative tumors who have an excellent prognosis with current treatment standards. Despite improvements in cancer outcomes within the CREATE‐X trial, the benefit of adjuvant capecitabine was less clear among clinically node‐negative patients with TNBC who failed to achieve pCR; risk reduction was notably lower in the node‐negative patients (HR 0.87, 95% CI 0.48–1.6) than in patients with pN1 disease (HR 0.54, 95% CI 0.36–0.83).^14^ The Adjuvant Paclitaxel and Trastuzumab (APT) trial evaluated small (≤2 cm), node‐negative, HER2+ breast cancers treated with up‐front surgery followed by adjuvant taxane and trastuzumab‐based therapy, demonstrating disease‐specific survival of 93% at 7‐years.^24^ Moreover, the ATEMPT trial demonstrated comparable safety profiles of adjuvant T‐DM1 to paclitaxel and trastuzamab within stage I HER2+ breast cancers; however, results from this phase II study found that women receiving T‐DM1 had a three‐fold greater risk of early discontinuation of therapy.^25^ Notably, toxicity of post‐neoadjuvant therapy should be weighed against its benefit, endorsing an individualized approach to decisions around NACT.

We found that the greatest change in utilization of NACT was seen in patients with T2 disease, of whom over two‐thirds received chemotherapy in the neoadjuvant setting. Among T2 patients who received NACT, at least half went on to breast conserving surgery, favoring its use in this cohort with relatively larger early breast cancers regardless of molecular subtype. Our findings also demonstrated that NACT was increasingly extrapolated outside the clinical trial eligibility to some patients with even very small tumors. Within the HER2+ and TNBC cohorts, patients with cT1a‐b cancers were twice as likely to receive NACT from 2010 to 2016. In contrast, for patients with high‐risk HR+/HER2‐breast cancer, only those with cT2 tumors saw an increase in NACT use during the study period, again suggesting that tumor downsizing may have been the driving force behind their management strategy.

The Alliance CALGB 40601 and 40603 trials demonstrated that NACT increased the rate of BCS eligibility by 23% and 14%, respectively, for HER2+ and TNBC patients.^26^, ^27^ However, real‐world data have more recently demonstrated a paradoxical relationship between NACT and receipt of contralateral prophylactic mastectomy (CPM). In the NCDB analysis conducted by Kantor et al., the authors found that patients who received NACT were 1.5 times as likely to undergo CPM.^28^ Similarly, Wapnir et al evaluated the California Cancer Registry and found that women who received NACT were twice as likely to undergo bilateral mastectomy, a difference that was largely attributed to rising trends of CPM on a national level.^23^ These results may not be unexpected in contemporary practice; the utility of pCR as a prognostic indicator and options for post‐neodjuvant treatment may overshadow the opportunity for surgical downstaging in recommendations for NACT.

To date, research evaluating the benefits of NACT has focused on prognostication, improving pathologic complete response rates, and determining eligibility for escalation of adjuvant therapies; however, the optimal role of NACT in the context of health equity and high‐value cancer care remains unknown.^29^ Findings from our study demonstrated that private versus public health insurance strongly correlated with receipt of NACT. As a result, chemotherapy in the preoperative setting may relate to health disparities. Robust literature has established that socioeconomic status (SES) is strongly associated with receipt of comprehensive breast cancer treatment; the care cascade associated with NACT (i.e., staging, breast MRI) may be burdensome for vulnerable cancer patients and/or factor into patient healthcare spending.^28^, ^29^, ^30^, ^31^, ^32^, ^33^ As NACT becomes increasingly offered to improve cancer‐specific survival, the breast oncology community must remain vigilant that health system biases do not allow sequence of chemotherapy to further contribute to health disparities or low‐value care.^32^, ^33^

Our study has several important limitations, and its results should be interpreted within the context of the available data. First, the NCDB does not include data related to cancer recurrence or disease‐specific survival which are critical oncologic outcomes. In addition, data available within national tumor registries limits our ability to delineate intent behind decisions for NACT. This is especially pertinent among women with HR+/HER2− breast cancers receiving NACT who did not have evidence of high recurrence risk genomic assay results, in whom decisions for NACT would have been driven by a variety of clinical and patient factors. Our study period pre‐dated publication of results from the TAILORx trial, which established that patients with OncotypeDX scores >25 benefitted from chemotherapy.^34^ From 2010–2016, decisions for NACT in patients with RS of 26–30 would have been driven by a variety of clinical and patient factors. Thus, our findings may underestimate trends of NACT use over time in this population. Of note, the NCDB does not clarify the timing of genomic assays. For the purpose of our study, we assumed that a high OncotypeDX score contributed to decisions for chemotherapy in the preoperative setting for all women with HR+/HER2‐ breast cancers who received NACT. Lastly, we included clinical T1‐2 tumors as a proxy for operable breast cancers, yet in reality, mammographic findings, breast‐to‐tumor ratio, and patient history contribute to decisions for preoperative chemotherapy.

## 5 CONCLUSION

Between 2010 and 2016, utilization of NACT increased by 2.5 times among women with high‐risk early breast cancer. This trend was moderated by molecular subtype and most notable among women with HER2+ and TNBC. Future research is needed to better understand multidisciplinary decisions for NACT and the implications for breast cancer patients.

## CONFLICT OF INTEREST

Dr. Hyslop and Ms. Thomas are consultants for AbbVie. Dr. Sammons is a consultant for Daiichi Sanko, Foundation Medicine, and Novartis. She receives research funding from Astra Zeneca, Eli Lilly, and Seattle Genetics.

## AUTHOR CONTRIBUTION

IP: Conceptualization, methodology, formal analysis, resources, writing—original draft, writing—review & editing. NBN: Methodology, formal analysis, data curation, visualization. SMT: Methodology, formal analysis. SS: Resources, writing—review & editing. RCB: Writing—review & editing. GAD: Writing—review & editing. TH: Writing—review & editing. CSM: Writing—review & editing. JKP: Writing—review & editing. LHR: Writing—review & editing. OMF: Writing—review & editing. ESH: Writing—review & editing. RAG: Conceptualization, methodology, writing—original draft, writing—review & editing, supervision, project administration.

## ETHICS

This retrospective study involving de‐identified data from human participants was conducted in accordance with the ethical standards of the 1964 Helsinki Declaration and its later amendments or comparable ethical standards. The institutional review board (IRB) at Duke University determined that our study did not need ethical approval, and our study was granted exempt status with a waiver of informed consent.

## PRESENTATION

This work was accepted for presentation at the 21st annual meeting of the American Society of Breast Surgeons (ASBrS) in Las Vegas, NV; cancelled due to COVID‐19.

## Data Availability

The National Cancer Data Base (NCDB) is a joint project of the Commission on Cancer (CoC) of the American College of Surgeons and the American Cancer Society. The CoC's NCDB and the hospitals participating in the CoC NCDB are the source of the de‐identified data used herein; they have not verified and are not responsible for the statistical validity of the data analysis or the conclusions derived by the authors. The data that support the findings of this study are available from the NCDB, but restrictions apply to the availability of these data, which were used under license for the current study and so are not publicly available. Data are, however, available from the authors upon reasonable request and with permission of the NCDB.
